# Flourishing in life in patients with Inflammatory Bowel Disease: The role of illness identity and health-related quality of life

**DOI:** 10.1177/13591053241260288

**Published:** 2024-07-25

**Authors:** Antonia Krömeke, Maor Shani

**Affiliations:** Osnabrück University, Germany

**Keywords:** flourishing in life, health-related quality of life, IBD, illness identity, social identification, subjective well-being

## Abstract

Amidst chronic challenges in Inflammatory Bowel Disease (IBD), including physical symptoms, emotional stress, and social constraints, this study aimed to elucidate how patients’ perceptions of their illness and its integration into their self-concept are related to their ability to flourish in life. We hypothesized that having a positive and integrative illness identity and social identification will predict higher flourishing, mediated by enhanced health-related quality of life (HRQoL). In an online survey with 244 German-speaking IBD adults (*M*_age_ = 36.62, 85% women), we found that lower engulfment (where the disease dominates one’s identity) predicted higher levels of flourishing, mediated by higher HRQoL. Enrichment, reflecting personal growth from illness, directly predicted higher flourishing, while stronger social identification predicted higher subjective well-being, but not flourishing. The results highlight the potential of fostering positive illness identities and social connections to enhance flourishing in individuals with IBD or similar chronic conditions.

Inflammatory Bowel Disease (IBD), an incurable autoimmune disorder that encompasses Ulcerative Colitis (UC) and Crohn’s Disease (CD), is marked by chronic intestinal inflammation, likely stemming from dysregulated inflammation, genetics, microbial, and environmental factors (Lemberg and Day, 2015; Mählmann et al., 2017). The rising incidence of IBD in Europe, with over 600,000 cases in Germany in 2010 (Hein et al., 2014), underscores the importance of addressing its multifaceted impact on patients’ lives.

IBD’s severity varies with periods of relapse and remission (Casellas et al., 2001), and persistent physical symptoms such as increased stool frequency, abdominal pain, weight loss, and extraintestinal issues (Knowles et al., 2018; Lemberg and Day, 2015) profoundly affect various facets of a patient’s life, highlighting the importance of psychological well-being in IBD management (Colonnello and Agostini, 2020). Physical discomfort can constrain social interactions and relationships. Patients may worry about body image, peer relationships, intimacy, and stigmatization (Hommel, 2013), and experience feelings of social isolation and inhibition (Jordi et al., 2021). Patients often struggle with the unpredictability of IBD, emotional turmoil, and strive to maintain a normal life despite the disease (Byron et al., 2020). Consequently, IBD severely impacts quality of life (Casellas et al., 2001; Knowles et al., 2018) and is also associated with psychological comorbidities like anxiety and depression (Graff et al., 2009). Patients may also experience maladaptive coping and adjustment problems related to lifelong treatment and feel worried and hopeless regarding their future, leading to maladaptive emotion-focused coping (Iglesias-Rey et al., 2013; Knowles et al., 2013).

Given the considerable psychosocial impact of IBD, exploring psychological mechanisms for interventions is crucial. These can enhance coping, resilience, and well-being, contributing to an integrated IBD management strategy that encompasses both physical and psychological health.

## HRQoL and flourishing in IBD patients

*Health-related quality of life* (HRQoL) encompasses physical, emotional, social, and psychological health perceptions and impacts on quality of life, focusing predominantly on health-related aspects (Knowles et al., 2018; Larussa et al., 2020; Tabibian et al., 2015). Factors like disease course and coping can significantly impact IBD patients’ HRQoL (Casellas et al., 2001). We focus on IBD-specific HRQoL because it more accurately reflects IBD patients’ health perceptions and functioning than generic measures, and directly addresses IBD-related issues including systemic symptoms, social function, and emotional state (Rose et al., 2000).

The concept *flourishing in life* captures the idea of optimal functioning, encompassing both hedonic and eudaimonic aspects of well-being (Kashdan et al., 2008; Mesurado et al., 2021; Seligman, 2011). Flourishing is conceptually linked to *subjective well-being* (SWB), which reflects individuals’ perceptions of their life experiences, including emotional and positive functioning (Diener et al., 2010). However, flourishing extends beyond merely assessing negative outcomes to promote positive health facets (Trompetter et al., 2019), aiming to capture a deep sense of purpose, positive relationships, engagement, and accomplishment (VanderWeele et al., 2019). It also transcends the mere absence of mental illness, encompassing positive emotional experiences, psychological resilience, and a sense of purpose and social contribution. In chronic illnesses like IBD, where patients face ongoing challenges and uncertainties, especially in affective and social domains, fostering flourishing becomes crucial (Edgar and Pattison, 2016).

While HRQoL is especially concerned with health status in physical, mental, emotional, and social domains, SWB and flourishing cover broader psychological life aspects, including emotional (e.g. happiness, contentment), psychological (e.g. self-acceptance, personal growth), and social well-being (e.g. social integration, contribution to society), on which health has only a modest impact (Holte et al., 2014). Recent studies have indicated that HRQoL positively predicts SWB in chronic illness patients (Maguire et al., 2021), yet research on SWB and flourishing in IBD patients remains limited (McCombie et al., 2013). High flourishing levels were linked to fewer physical complaints, reduced healthcare usage, and increased productivity (Diener et al., 2010; Huppert, 2009), emphasizing its importance in managing chronic conditions like IBD. Accordingly, we hypothesize that better HRQoL correlates with an overall enhancement in well-being, as individuals engaging in satisfying activities and lifestyles likely perceive their physical and psychological health as high. Additionally, the emotional and social HRQoL domains, which are crucial in IBD, promote an emphasis on emotion-focused coping mechanisms (Iglesias-Rey et al., 2013; Knowles et al., 2013), which may enhance social support and relationships, thereby elevating well-being and flourishing. Therefore, our first hypothesis is:

*H1: HRQoL will be positively associated with flourishing.*

## The role of illness identity and social identification in HRQoL and flourishing

Identity formation, a crucial aspect of human development, refers to how individuals perceive themselves and are perceived by others within societal norms, including personal traits, roles, values, and group affiliations that shape self-conception and presentation (Jenkins, 2014). *Illness identity*, referring to the degree to which a chronic illness integrates into one’s identity and how it defines one’s self and their relationship to others (Oris et al., 2016), involves four dimensions: engulfment (where the disease dominates identity), rejection (denial of illness identity), acceptance (incorporation of the illness while striving for normalcy), and enrichment (using the illness for personal growth). Engulfment and rejection signify maladaptive integration, whereas acceptance and enrichment represent more adaptive approaches to integrating illness (Oris et al., 2016, 2018).

Studies in chronic patients have shown that identity processes and perceptions related to one’s illness significantly impact overall psychological well-being (Van Bulck et al., 2019). Engulfment and rejection were found to correlate with maladaptive outcomes, while acceptance and enrichment were associated with more adaptive psychological functioning (Luyckx et al., 2015; Oris et al., 2018). Notably, illness identity dimensions are strongly related to social and emotional domains of HRQoL, which are vital in IBD (Oris et al., 2018; Shneider et al., 2024). In the context of IBD, illness identity significantly influences adherence to treatment, mental health, HRQoL, and coping strategies (Peters and Brown, 2022; Rassart et al., 2023). Rassart et al. (2023) found HRQoL in IBD patients to be positively related to acceptance and negatively to engulfment, while depression was negatively related to acceptance but positively related to both engulfment and rejection. In a German sample of IBD patients, both engulfment and rejection were negatively associated with HRQoL, while acceptance (but not enrichment) demonstrated a positive relationship with HRQoL (Thomann et al., 2023). Considering these theoretical underpinnings and preliminary evidence, we would expect illness identity to predict HRQoL in a sample of IBD patients:

*H2a: The illness identity domains of acceptance and enrichment will positively predict HRQoL.*
*H2b: The illness identity domains of engulfment and rejection will negatively predict HRQoL.*

The role of illness identity in flourishing has not yet been studied among IBD patients, or, to our knowledge, in other chronic conditions (Tabibian et al., 2015). Research indicates that higher levels of identity enrichment and lower levels of engulfment are crucial for adaptive coping, treatment adherence, and aspects of eudaimonic well-being. Such enrichment, reflecting the perception of illness as a source of personal growth, contrasts with engulfment, where illness overwhelms personal identity (Commissariat et al., 2023; Rassart et al., 2023). Illness acceptance was also linked to eudaimonic well-being, encompassing personal growth and self-acceptance, in several chronic conditions, suggesting its potential benefit for both the physical and social facets of quality of life (Szcześniak et al., 2020; Wysocki et al., 2023). Considering the psychological difficulties in IBD management, positive and integrative illness identity is expected to pave an adaptive coping path for IBD patients and to be particularly relevant to social and emotional aspects of HRQoL as well as to more general well-being constructs. The enrichment and engulfment dimensions may be particularly important for self-fulfillment aspects of well-being captured by flourishing. Specifically, engulfment potentially leads to a diminished sense of self beyond the illness, while enrichment allows individuals to derive personal growth from their illness experience, which is a particularly important prerequisite for flourishing (Mesurado et al., 2021; Trompetter et al., 2019). This leads to our third hypothesis:

*H3a: Acceptance and enrichment will be positively associated with flourishing.*
*H3b: Engulfment and rejection will be negatively associated with flourishing.*

In addition to the four domains of illness identity, we examined the role of social identification, based on the concepts of social identity and social categorization (Aron et al., 1992). While illness identity captures the nature of integration of one’s illness into their self-concept, social identification refers to personal meaning derived from ingroup membership (Tajfel and Turner, 2004), and in this context, the extent to which an IBD patient feels close to or identifies with other IBD patients as a social group. Although extensive research highlights the benefits of social identification for health behaviors and outcomes (De Hoog and Pat-El, 2024), studies on identification within specific condition groups remain scarce. Cameron et al. (2018) showed that within chronic illness self-management programs, group identification enhanced self- and group-efficacy, leading to improved health. Similarly, research has underscored the psychosocial advantages of belonging to groups providing support and fostering a positive social identity (Haslam et al., 2009). Studies among chronically ill students have shown the value of illness-related social identities in managing the illness and engaging in life (Spencer and Almack, 2023). Given that identifying with relevant groups can boost perceived control, self-efficacy, and support (Haslam et al., 2009; Junker et al., 2019), we hypothesized that IBD patient identification would offer essential coping resources, promoting better HRQoL and flourishing:

*H4a: Social identification will positively predict HRQoL*
*H4b: Social identification will be positively associated with flourishing.*

Finally, we considered the mechanism linking illness identity to flourishing. Specifically, higher acceptance and enrichment by one’s illness identity, parallel to lower feelings of rejection and engulfment, may lead to more effective coping and engagement in positive health-related behaviors, directly increasing HRQoL and consequently fostering flourishing. Previous studies found that illness identity predicted HRQoL among IBD patients (Peters and Brown, 2022; Rassart et al., 2023), and that HRQoL mediated the relationship between illness perceptions, such as optimism, and SWB (Shang et al., 2018). We suggest extending these findings across illness identity dimensions, aiming to demonstrate that positively integrating one’s illness into the self-concept can directly improve patients’ HRQoL perceptions, which then affect their potential for flourishing. While lower HRQoL may contribute to reduced flourishing as patients struggle with the implications of their illness, higher HRQoL may enable greater flourishing as patients feel less hindered in pursuing well-being. Thus, HRQoL may translate the potential positive effects of illness identity and identification into overall life satisfaction. Based on this rationale, we propose the following final hypothesis:

*H5a: The relationship between illness identity dimensions (engulfment, rejection, acceptance, and enrichment) and flourishing will be mediated by HRQoL.*
*H5b: The relationship between social identification and flourishing will be mediated by HRQoL.*

## The current study

**Figure 1.**
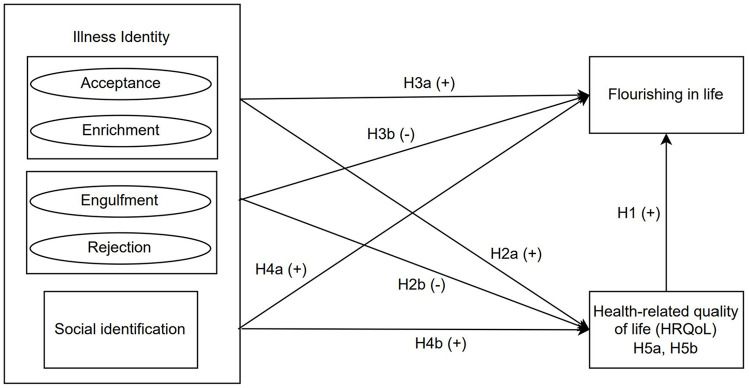
Conceptual model. H5a and H5b are indirect effects linking illness identity and social identification, respectively, with flourishing in life. H: Hypothesis.

Figure 1 presents the theoretical model delineating the hypothesized relationships and the anticipated directions of associations. This study bridges the literature gap on illness identity and flourishing in IBD management within a sample of adults with IBD in Germany, seeking to elucidate the interplay between the four domains of illness identity and social identification with flourishing, with an emphasis on the mediating role of HRQoL. While evaluating our hypotheses, we acknowledge the exploratory nature of our research concerning these novel associations, attributed to existing gaps in the literature. Consequently, although our primary focus is on the dynamics between illness identity, HRQoL, and flourishing, we also exploratively assess SWB as an additional outcome in our statistical model. Given the similarities and potential overlaps between SWB and flourishing (Diener et al., 2010; Huppert, 2009), our aim is to investigate how results for flourishing may align with or diverge from those for SWB, without establishing definitive hypotheses.

## Materials and methods

### Participants and procedure

This research employed a cross-sectional design, collecting data through an online survey from adult IBD patients. Participants met the following criteria: (1) self-reported diagnosis of CD or UC, (2) aged between 18 and 65, and (3) proficient in German. A non-probabilistic sampling method was used. Study invitations, along with detailed flyers, were distributed via IBD-focused social media channels. Completing the survey took approximately 15 minutes, and participation was voluntary, incentivized by a raffle for a 100-euro prize to encourage participation. The study protocol was approved by the Ethics Committee of the authors’ institution, and all respondents provided informed consent online.

Detailed information about the sample is provided in the Online Supplementary Materials (OSM). An a priori Monte Carlo power analysis for indirect effects was conducted in R using the shiny application (Schoemann et al., 2017). We targeted a power of 0.80 and a significant criterion of α = 0.05 for detecting medium effect sizes. The analysis indicated that a sample size of 228 participants would yield a power of 1− β = 0.84 (95% CI [0.80, 0.87]). The original dataset comprised 258 completed questionnaires. After excluding non-IBD respondents, 10 cases were deleted, leaving 248 cases. Additionally, we removed three cases involving participants under 18 years of age and one participant with a very short processing time (124 seconds) and 98% missing values. Consequently, the final dataset included *N* = 244 cases, satisfying the requirements outlined in the a priori power analysis. The sample was predominantly female (84.8%), with an average age of 36.62 (SD = 10.2). This gender distribution is consistent with previous findings that reported a higher incidence of IBD in women than men (Hein et al., 2014).

Pertaining to illness characteristics, 75.4% had UC, 21.3% had CD, and 3.3% reported suffering from other forms of IBD, closely matching the distribution of IBD types within the German population (Hein et al., 2014). Further disease-related details about the sample are accessible in Table S2 (OSM).

### Measures

All instruments were either originally developed in German, used as validated German translations, or translated into German by multilingual individuals through translation and back-translation procedures. Scores for scales with multiple items were calculated by determining the arithmetic mean of all valid items, after inverting the scores for reversed items. Higher scores indicated greater expression of the constructs measured.

The German Short Inflammatory Bowel Disease Questionnaire (SIBDQ) was used to evaluate *HRQoL* (Rose et al., 2000). Designed to quantify IBD-related challenges, such as bowel symptoms and emotional issues, this instrument comprises ten items referring to experiences within the 2 weeks prior to response. Of these items, eight are assessed on a 7-point scale (1 = *always*, 7 = *never*), with an example being: “During the last 2 weeks, how frequently has fatigue been problematic for you?” The remaining two items utilized a different 7-point scale (1 = *Distinct problems*, 7 = *no problems*), illustrated by: “Have your bowel issues caused difficulties with recreational or sporting activities you wished to engage in the past 2 weeks?” The questionnaire demonstrated high internal consistency with a Cronbach’s α = 0.86.

*Illness Identity* was measured using the Illness Identity Questionnaire (IIQ; Oris et al., 2016), which covers four dimensions: engulfment (eight items), enrichment (eight items), acceptance (six items), and rejection (five items). Participants expressed their level of agreement with each item on a 5-point Likert scale ranging from 1 (*completely disagree*) to 5 (*completely agree*), thereby evaluating the extent of IBD integration into their identity. Considering its initial development for diabetes and limited application to IBD (Peters and Brown, 2022; Rassart et al., 2023), we conducted a confirmatory factor analysis (a detailed analysis and table with factor loadings are provided in OSM). The instrument demonstrated strong internal consistency for engulfment (α = 0.906), enrichment (α = 0.879), and acceptance (α = 0.856), while rejection showed satisfactory consistency (α = 0.626).

*Social identification* was assessed using the Inclusion of the Other in the Self (IOS) scale (Aron et al., 1992), a single-item graphical tool capturing perceived closeness to a group, in this instance, the IBD community. Utilized in the context of chronic illness, the IOS evaluates communal coping, which is linked to improved psychological well-being and physical health (Helgeson et al., 2017; Shani et al., 2022). Participants chose from seven diagrams that depicted varying degrees of overlap between two circles, symbolizing themselves and the IBD community. The scale ranges from 1 (*no overlap*) to 7 (*almost complete overlap*).

The Flourishing Scale’s German version (FS-D; Esch et al., 2013), based on the original scale (Diener et al., 2010), was employed to measure *flourishing in life*. Participants rated eight items on a scale from 1 (*completely disagree*) to 7 (*completely agree*), for example: “I lead a purposeful and meaningful life.” The FS-D demonstrated high consistency (α = 0.896) across patient groups.

The World Health Organization Well-Being Index (WHO-5; World Health Organization, 2013) assessed *SWB* using a 5-item scale, with responses from 0 (*at no time*) to 5 (*all the time*), covering the preceding 2 weeks (e.g. “I have felt calm and relaxed”). The scale showed high internal consistency (α = 0.88).

The German Inflammatory Bowel Disease Activity Index (GIBDI; Hüppe et al., 2018) uses a 13-item index to distinguish factors for UC and CD. For UC, scoring considered the frequency of loose or bloody stools, whereas CD included additional factors like fever and weight. Scores, computed following Hüppe et al. (2018) for UC and CD, classified disease activity from remission (0–3) to severe (12 and above). The GIBDI was not applied to participants with indeterminate colitis, stoma, or pouch, due to questionnaire incompatibility.

Finally, illness-related variables recorded were IBD variant (UC or CD) and disease duration. Demographic data comprised age, gender, education level, occupation, and socioeconomic status (SES), measured on a subjective ladder from 1 to 10, with 10 denoting high SES.

### Statistical analysis

The complete dataset and analysis syntax are available on the study’s OSF page: https://osf.io/v2pem. Little’s test was non-significant, χ^2^(161) = 181.64, *p* = 0.127, indicating randomness of missing data, which was removed listwise. Descriptive statistics were calculated for data examination. All tests were two-tailed with a significance level set at *p* < 0.05.

Correlational and path analyses investigated the study’s hypotheses. Bivariate Pearson correlations assessed relationships between key variables, facilitating the examination of hypotheses H1, H3, and H4b. Path analysis in Mplus, considering illness identity domains and social identification as predictors, HRQoL as a mediator, and flourishing and SWB as outcomes, tested direct (H2, H4a) and indirect (H5) effects. Bootstrapped, bias-corrected 95% Confidence Intervals (CIs) with 5000 resamples were used for indirect effects. Despite slight deviations from normality in flourishing and SWB, this did not limit the analysis due to the nature of bootstrapped CIs not requiring normal distribution (Hayes, 2018).

Finally, the robustness of findings was further validated in a modified path model that included sociodemographic and illness-related variables that showed correlation with outcomes as covariates. Detailed results are available in the OSM and are briefly summarized below.

## Results

First, consistent with H1, HRQoL was positively and strongly related to both flourishing and SWB. The relationship between flourishing and SWB was also strong and positive. Second, consistent with H3, all four illness identity dimensions significantly correlated with flourishing and SWB. Specifically, higher SWB and flourishing were both strongly associated with lower engulfment, but more weakly related to lower rejection. Higher acceptance was positively and moderately associated with both higher flourishing and SWB, whereas enrichment showed moderate-to-high correlations with higher flourishing and SWB. Third, social identification had a weak but positive correlation with both flourishing and SWB, supporting H4b.

**Figure 2.**
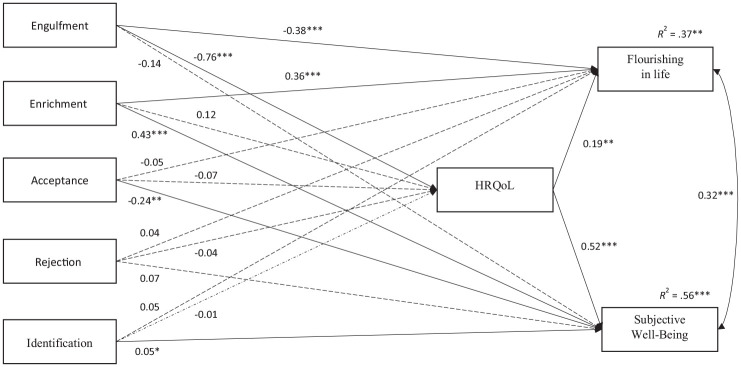
Diagram of path model predicting flourishing and subjective well-being. *N* = 243. Path coefficients were unstandardized estimates. Dashed lines represent nonsignificant effects. Covariances between predictors are included in the model but are not shown graphically. HRQoL: health-related quality of life. *p < 0.05. **p < 0.01. ***p < 0.001.

Path analysis was used to examine direct and indirect effects. Figure 2 displays the statistical model and path coefficients. Multicollinearity was not detected (all VIF values were lower than 2.05). Consistent with H1, higher HRQoL predicted higher Flourishing and SWB. Higher HRQoL was significantly predicted only by lower engulfment, providing partial support for H2b. Identification with other IBD patients did not significantly predict HRQoL, leading to rejection of H4a. The analysis also identified significant direct effects: higher flourishing was predicted by both lower engulfment and higher enrichment. Furthermore, higher SWB was significantly predicted by higher enrichment and higher identification. Despite acceptance showing a positive (albeit weak) correlation with SWB, when including all illness identity dimensions as parallel predictors in the path analysis, higher acceptance significantly predicted lower SWB, suggesting a potential suppression effect.

The mediating role of HRQoL (H5) was inspected through bootstrapped CIs for indirect effects. Detailed results are presented in Table S6 (OSM). HRQoL significantly mediated the relationships between engulfment and flourishing (*B* = −0.14, SE = 0.05, 95% CI [−0.258, −0.057]), and between engulfment and SWB (*B* = −0.40, SE = 0.06, 95% CI [−0.518, −0.303]). Essentially, higher engulfment was associated with lower HRQoL, which was then associated with lower flourishing and SWB. Thus, the results provided partial support for H5.

The robustness of these findings was further assessed by incorporating sociodemographic (age, gender, SES) and disease-related variables (IBD variant, disease duration, GIBDI) as controls. These additional analyses yielded broadly similar results, which are detailed in the OSM.

## Discussion

Flourishing, marked by a sense of purpose, strong social connections, and an optimistic outlook on life (Mesurado et al., 2021; Seligman, 2011; VanderWeele et al., 2019), remains underexplored within the IBD patient demographic. This study sought to determine whether domains of illness identity, crucial in the emotional and social dimensions of HRQoL (Shneider et al., 2024), significantly predict flourishing in individuals with IBD. The results reveal a significant association between HRQoL and both flourishing and SWB, endorsing H1 (see Figure 1) and the concept that physical and mental health is fundamental to well-being (Huppert, 2009). Associations across all four domains of illness identity with these outcomes were observed, largely affirming H3. However, HRQoL was found to mediate only the relationship between engulfment and both flourishing and SWB, offering partial validation of H2 (illness identity’s prediction of HRQoL) and H5 (HRQoL’s mediation of effects on flourishing), with the domain of enrichment directly predicting flourishing in our analysis. Contrary to H4, elevated social identification was linked only to higher SWB, not flourishing. This section delves into these principal findings and their theoretical and practical implications.

Our results highlight the pivotal roles of two illness identity domains, enrichment and engulfment, in predicting flourishing among IBD patients, largely corroborating the hypotheses. Yet, the indirect influence of illness identity on flourishing through HRQoL was only observed for engulfment. Lower levels of engulfment were associated with decreased flourishing and SWB, both directly and via a reduction in IBD-specific HRQoL. This aligns with literature suggesting individuals with high engulfment may feel overwhelmed by their disease, leading to a loss of personal identity beyond their patient status (Luyckx et al., 2015; Rassart et al., 2023). Engulfment significantly detracts from flourishing by causing individuals with IBD to view their lives primarily through the lens of their illness, limiting their perception of identity and life’s possibilities. The dominance of illness over identity can diminish well-being and impede the ability to thrive, consequently impacting HRQoL and the potential for flourishing (Luyckx et al., 2015; Oris et al., 2016, 2018). The strong direct and indirect effects of engulfment might be attributed to hypervigilance, characterized by heightened alertness to threats and excessive protectiveness that can provoke stress and diminish resilience (Rollman, 2009). In the context of IBD, this could be evidenced as visceral hypervigilance, or an increased focus on and negative emotional response to abdominal symptoms (Atanasova et al., 2022). Hypervigilance may either result from engulfment or act as a mediator between engulfment, HRQoL, and flourishing. Future research should further explore hypervigilance’s role in the management of IBD, particularly in relation to engulfment.

The findings further highlight the significance of enrichment, where a sense of personal growth and positive transformation arising from illness directly predicted higher flourishing and SWB, independent of HRQoL’s mediating role. When individuals integrate their experiences into a broader narrative of self-development and resilience (Oris et al., 2016, 2018), their identity may promote a more holistic sense of well-being, facilitating flourishing. These results align with prior research linking stronger enrichment to better HRQoL and greater life satisfaction among IBD patients (Rassart et al., 2023), as well as with better quality of life across various chronic conditions (Shneider et al., 2024). Individuals who view their illness as an opportunity for enrichment might experience personal development through facing its challenge, aligning with the concept of *post-traumatic growth* (Hamama-Raz et al., 2021). This suggests adversity can spur significant personal evolution and a reassessment of life’s values (Tedeschi and Calhoun, 2004), emphasizing the empowering dimension of enrichment where patients discover positive meaning in their condition, promoting a growth-focused identity. In summary, our findings suggest that the dynamic processes of engulfment and enrichment are central to understanding how illness identity influences flourishing. Engulfment constrains the self, binding individuals to a narrative of limitation, while enrichment expands the self, incorporating the illness into a narrative of growth and resilience (Van Bulck et al., 2019).

Our fourth hypothesis, rooted in social identity theory, suggested that identification would indirectly predict flourishing, through elevated HRQoL. Yet, identification demonstrated only weak positive associations with SWB and flourishing, and not with HRQoL, directly predicting higher SWB in the uncontrolled model alone. This is partly in line with theories on social identity and health, advocating that strong group connectivity can offer emotional support, practical advice, and stress resilience (Haslam et al., 2009). Nevertheless, these relatively mild associations diverge slightly from recent studies indicating that interventions aimed at bolstering social identification exerted moderate-to-strong effects on health and well-being (Steffens et al., 2021). The subdued associations between social identification and flourishing may stem from flourishing’s complex nature, which may depend more on individual factors like resilience, growth, and self-efficacy than on external influences such as social identification. Although identification was a predictor for SWB, it did not predict flourishing. SWB encompasses cognitive and affective evaluations of one’s life (Diener et al., 2010), whereas flourishing covers a broader spectrum, including emotional well-being, psychological functioning, personal development, and life meaning (Mesurado et al., 2021; VanderWeele et al., 2019). This suggests that while group identification can improve life satisfaction (as indicated by SWB), it may not necessarily enhance optimal psychological functioning (as captured by flourishing). Future studies could further explore these dynamics by focusing on specific social networks, like IBD support groups, which might have a more pronounced impact on psychological outcomes by bolstering peer support as a coping mechanism (Haslam et al., 2009; Junker et al., 2019).

In our study, neither acceptance nor rejection significantly predicted flourishing. These domains could have a lesser impact on flourishing, potentially because they stabilize illness identity without transforming it. They may be foundational for adaptation but not inherently driving growth. Previous research has highlighted the mixed implications of rejection on psychosocial health (Shneider et al., 2024), with some individuals possibly leveraging rejection as a form of disengagement coping, which could have neutral or potentially positive effects on well-being. The adverse impact of rejection on treatment adherence and self-management suggests its influence on HRQoL may be mediated more significantly over time (Knowles et al., 2013, 2018). Conversely, stronger acceptance did not emerge as a predictor for flourishing or SWB through HRQoL. This finding is consistent with some literature showing minimal links between acceptance and patient-reported outcomes, but diverges from studies associating higher acceptance with improved quality of life and adaptive psychological functioning (Rassart et al., 2023; Shneider et al., 2024). The influence of acceptance on such outcomes could be moderated by self-care behaviors, implying that acceptance within illness identity may boost HRQoL and psychological well-being by encouraging self-care practices. This potential pathway warrants further investigation in future studies.

### Limitations and future research

Although our study tested a causal model, the cross-sectional design limits the ability to draw causal or temporal conclusions. The results support the proposed pathway involving the engulfment dimension of illness identity; however, the possibility of bidirectional relationships or alternative explanations remains. For example, HRQoL could influence how IBD patients perceive their illness identity, which in turn could affect their well-being. Likewise, the relationship between HRQoL and well-being is likely to be more dynamic and reciprocal than the unidirectional approach suggested by our statistical model. To further substantiate these findings, future research should utilize longitudinal designs and experience sampling methods to provide a more comprehensive understanding of these relationships.

Subsequent research should examine additional mechanisms in addition to HRQoL, such as personality traits, coping strategies, perceived stigma, and support networks, that may also play significant roles. Moreover, the impact of social identification within the context of IBD warrants more in-depth exploration. Factors like over-identification and hypervigilance, which may be associated with engulfment, could hinder personal development and psychological well-being (Atanasova et al., 2022). With regard to social identification, the influence of specific patient ingroup identification, like local support groups or online communities, was overlooked in this study and should be considered in future studies.

Moreover, while our IBD sample was large and diverse, its size was not optimal for fully leveraging the benefits of Structural Equation Modeling with latent constructs, though path analysis remains valid for exploring psychological mechanisms (Valente et al., 2017). The potential for self-selection bias may affect the generalizability of our findings. Our sampling strategy, targeting German-speaking individuals, might have attracted participants particularly attuned to their illness identity, possibly biasing our results. Considering the potential differences in HRQoL between UC and CD patients, and across genders (Casellas et al., 2001; Tabibian et al., 2015), future research should aim to replicate our findings in diverse samples across different cultural contexts.

Finally, our focus on flourishing could be expanded to include other indicators of a “good life” such as meaning in life and psychologically rich life (Oishi and Westgate, 2022), along with eudaimonic well-being. Eudaimonic well-being focuses on fulfilling one’s potential and living authentically in pursuit of autonomy, personal goals, life purpose, and self-realization. These concepts more comprehensively represent the intricacies of well-being and flourishing (Kashdan et al., 2008).

### Practical implications

While acknowledging the limitations of our preliminary study and its limited generalizability, the results suggest potential avenues for interventions to enhance life satisfaction among IBD patients, addressing a noted gap in the literature on managing social and psychological challenges in IBD and similar conditions (Byron et al., 2020). Despite satisfactory care, many patients refrain from discussing psychological and social aspects of their illness with healthcare providers, highlighting a need for more comprehensive care (Larussa et al., 2020). Moreover, the inclination toward non-conventional therapies and psychological support among patients signals a call for innovative care strategies (Hein et al., 2014). In line with the recognized need for such strategies, our results indicate that a patient-centered, flourishing-focused approach could beneficially impact HRQoL and well-being (VanderWeele et al., 2019). Fostering an environment where patients are encouraged to find meaning and growth in their experiences could enhance HRQoL and, subsequently, flourishing.

Furthermore, our study offers initial evidence on the importance of addressing illness identity in promoting patients’ flourishing. This aligns with existing research emphasizing the beneficial role of positive illness identity states in chronic condition management (Rassart et al., 2023; Shneider et al., 2024). Tailored interventions that help patients integrate their diagnosis into their self-concept could encourage illness-related enrichment and reduce feelings of engulfment, potentially aiding in maintaining a balanced identity (April et al., 2022). In this context, interventions such as compassion and acceptance-based therapies could be effective in facilitating integrative illness identity (Trindade et al., 2016). Finally, the role of social identification, though not as pronounced as other factors, warrants further exploration, especially in designing community-based support and engagement interventions, integrating self-help and social support in holistic treatments (Steffens et al., 2021).

These implications, while promising, are preliminary and call for further research to substantiate the efficacy of such interventions. We recommend cautious implementation in the framework of pilot programs or ongoing evaluations. As IBD management evolves, incorporating research evidence into practice could significantly enhance the quality of life for patients, encouraging not just survival, but also flourishing in life.

## Supplemental Material

sj-docx-1-hpq-10.1177_13591053241260288 – Supplemental material for Flourishing in life in patients with Inflammatory Bowel Disease: The role of illness identity and health-related quality of life Supplemental material, sj-docx-1-hpq-10.1177_13591053241260288 for Flourishing in life in patients with Inflammatory Bowel Disease: The role of illness identity and health-related quality of life by Antonia Krömeke and Maor Shani in Journal of Health Psychology
