# Supplementary material for: Flourishing in life in patients with Inflammatory Bowel Disease: The role of illness identity and health-related quality of life
Source: J Health Psychol. 2024 Jul 25;30(5):1089–103. doi: 10.1177/13591053241260288 (PMC11977818; doi:10.1177/13591053241260288)
Supplement: sj-docx-1-hpq-10.1177_13591053241260288 – Supplemental material for Flourishing in life in patients with Inflammatory Bowel Disease: The role of illness identity and health-related quality of life [file sj-docx-1-hpq-10.1177_13591053241260288.docx]

**Flourishing in Life in Patients with Inflammatory Bowel Disease:
The Role of Illness Identity and Health-Related Quality of Life**

**Online Supplementary Materials**

| **Table S1**  *Descriptive Sociodemographic Characteristics of the Participants (N =244)* | |
| --- | --- |
| **Age** |  |
| Mean (*SD*), years | 36.62 (10.2) |
| Range | 18-65 |
| **Gender** |  |
| Female % (*n*) | 84.8 (207) |
| Male % (*n*) | 14.8 (36) |
| Other % (*n*) | 0.4 (1) |
| **Subjective social status** |  |
| Mean (SD) | 5.27 (1.9) |
| Range | 1-10 |
| **Employment status** |  |
| Pupil | 0.8 (2) |
| Apprentice | 2.5 (6) |
| Student | 5.3 (13) |
| Employee | 57.8 (141) |
| Self-employed or freelancer | 3.3 (8) |
| Pensioner | 7.8 (19) |
| Other | 22.5 (55) |
| **Level of education** |  |
| No degree % (*n*) | 0.4 (1) |
| Main school diploma % (*n*) | 11.9 (29) |
| High school diploma % (*n*) | 35.7 (87) |
| Baccalaureate % (*n*) | 32.4 (79) |
| University degree (Bachelor, Master) % (*n*) | 19.3 (47) |
| PhD % (*n*) | 0.4 (1) |
| **Table S2**  *Descriptive Disease-Related Information of Participants* | |
| **Disease type** (*N* = 244) |  |
| Crohn’s % (*n*) | 21.3 (52) |
| Colitis % (*n*) | 75.4 (184) |
| Other % (*n*) | 3.3 (8) |
| **Disease status** (*N* = 237) |  |
| Remission % (*n*) | 17.3 (41) |
| Mild % (*n*) | 34.2 (81) |
| Moderate % (*n*) | 30.0 (71) |
| Severe % (*n*) | 18.6 (44) |
| **Disease duration** (*N* = 241) |  |
| Mean (*SD*), years | 9.36 (9.2) |
| Range | 0-44 |
| **Medication** (*N* = 389) |  |
| Prednisolone, Prednisone % (*n*) | 12.3 (48) |
| Budesonide % (*n*) | 8.5 (33) |
| Mesalazine % (*n*) | 29.6 (115) |
| Sulfasalazine % (*n*) | 1.5 (6) |
| Azathioprine % (*n*) | 5.4 (21) |
| 5-mercaptopurine % (*n*) | 0.5 (2) |
| TNF-alpha inhibitors % (*n*) | 22.1 (86) |
| Other % (*n*) | 12.9 (50) |
| Not known % (*n*) | 0.3 (1) |
| No medication % (*n*) | 6.9 (27) |

**Confirmatory Factor Analysis on the Illness Identity Questionnaire**

*Illness Identity* was measured using the Illness Identity Questionnaire (IIQ) (Oris et al., 2016). It encompasses four dimensions: engulfment (8 items), enrichment (8 items), acceptance (6 items), and rejection (5 items). Participants rated their agreement with each item on a five-point Likert scale from 1 (*completely disagree*) to 5 (*completely agree*). In this context, the degree of IBD integration into one's identity was evaluated. Given its original use was for diabetes and limited application for IBD (Peters, 2021; Rassart et al., 2023), we performed a confirmatory factor analysis (CFA) using Mplus 8.3. The four-factor model resulted in an unsatisfactory model fit: χ^2^(318) = 740.605, p < 0.001; RMSEA = 0.074; CFI = 0.867; TLI = 0.853; SRMR = 0.071. After addressing modification indices, we improved the model fit by adding five error correlations among related items within a single factor (items 3 - 4, 3 - 5, 13 - 15, 21 - 23, 23 - 27). The final four-factor model was found to be acceptable according to Hopwood and Donnellan’s (2010) model fit guidelines: χ*2*(313) = 593.196, p < 0.001; RMSEA = 0.061; CFI = 0.91207; TLI = 0.901, SRMR = 0.066. Table S1 (OSM) provides the items of the IIQ and their standardized factor loadings within the final four-factor model. Strong internal consistency was shown for engulfment (α = .906), enrichment (α = .879), and acceptance (α = .856). Rejection displayed satisfactory consistency (α = .626).

**Table S3**

*Standardized Factor Loadings for the Final Four Factor Model of Illness Identity*

| Rejection |  |
| --- | --- |
| 1. I refuse to see my bowel disease as part of myself. | .826 |
| 2. I’d rather not think of my bowel disease. | .430 |
| 3. I never talk to others about my bowel disease. | .229 |
| 4. I hate being talked to about my bowel disease. | .336 |
| 5. I just avoid thinking about my bowel disease. | .120 |
| Acceptance |  |
| 6. My bowel disease simply belongs to me as a person. | .837 |
| 7. My bowel disease is part of who I am. | .680 |
| 8. I accept being a person with bowel disease. | .837 |
| 9. I am able to place my bowel disease in my life. | .783 |
| 10. I have a clear picture or understanding of my bowel disease. | .494 |
| 11. I have learned to accept the limitations imposed by my bowel disease. | .494 |
| Engulfment |  |
| 12. My bowel disease dominates my life. | .841 |
| 13. My bowel disease has a strong impact on how I see myself. | .635 |
| 14. I am preoccupied with my bowel disease. | .738 |
| 15. My bowel disease influences all my thoughts and feelings. | .643 |
| 16. My bowel disease completely consumes me. | .806 |
| 17. It seems as if everything I do, is influenced by my bowel disease. | .719 |
| 18. My bowel disease prevents me from doing what I would really like to do. | .696 |
| 19. My bowel disease limits me in many things that are important to me. | .792 |
| Enrichment |  |
| 20. Because of my bowel disease, I have grown as a person. | .793 |
| 21. Because of my bowel disease, I know what I want out of life. | .590 |
| 22. Because of my bowel disease, I have become a stronger person. | .887 |
| 23. Because of my bowel disease, I realize what is really important in life. | .583 |
| 24. Because of my bowel disease, I have learned a lot about myself. | .649 |
| 25. My bowel disease has brought me closer to my friends and family. | .546 |
| 26. Because of my bowel disease, I have learned to work through problems and not just give up. | .743 |
| 27. Because of my bowel disease, I have learned to enjoy the moment more. | .605 |

*Note. N* = 240. For CFA, all factor loadings are statistically significant at *p* < .001.

**Table S4**

*Descriptive Statistics for Main Variables*

| Variable | *N* | *M* | *SD* | Skewness | Kurtosis |
| --- | --- | --- | --- | --- | --- |
| Flourishing | 244 | 4.98 | 1.11 | -0.41 | -0.50 |
| Subjective Well-Being | 244 | 2.94 | 1.10 | 0.34 | -0.89 |
| HRQoL | 244 | 3.86 | 1.21 | 0.06 | -0.51 |
| Acceptance | 244 | 3.35 | 0.93 | -0.21 | -0.76 |
| Enrichment | 244 | 3.20 | 0.94 | -0.16 | -0.64 |
| Engulfment | 244 | 3.13 | 0.98 | -0.15 | -0.63 |
| Rejection | 244 | 2.65 | 0.79 | 0.07 | -0.44 |
| Ingroup Identification | 243 | 4.15 | 1.94 | 0.01 | -1.15 |

*Note.* HRQoL= health-related quality of life.

**Table S5**

*Means, Standard Deviations and Correlations among Study Variables*

| Measure | 1 | 2 | 3 | 4 | 5 | 6 | 7 | 8 |
| --- | --- | --- | --- | --- | --- | --- | --- | --- |
| 1. Flourishing | - |  |  |  |  |  |  |  |
| 2. SWB | .68^***^ | - |  |  |  |  |  |  |
| 3. HRQoL | .45^***^ | .68^***^ | - |  |  |  |  |  |
| 4. Engulfment | -.50^***^ | -.48^***^ | -.63^***^ | - |  |  |  |  |
| 5. Enrichment | .38^***^ | .36^***^ | .16^*^ | -.15^*^ | - |  |  |  |
| 6. Acceptance | .30^***^ | .19^**^ | .20^**^ | -.29^***^ | .65^***^ | - |  |  |
| 7. Rejection | -.21^**^ | -.16^*^ | -.22^**^ | .31^***^ | -.32^***^ | -.48*^**^ | - |  |
| 8. Ingroup Identification | .19^**^ | .18^**^ | .05 | -.07 | .29^***^ | .25^***^ | -.20^**^ | - |
| Mean | 4.98 | 2.94 | 3.86 | 3.13 | 3.20 | 3.35 | 2.65 | 4.15 |
| *SD* | 1.11 | 1.01 | 1.21 | 0.98 | 0.94 | 0.93 | 0.79 | 1.94 |

*Note. N* = 244. HRQoL= health-related quality of life. SWB = Subjective Well-Being.

**p* < .05. ***p* < .01. and ****p* < .005.

**Gender and IBD type differences**

Regarding gender differences, males reported higher levels of rejection (*M* = 2.92, *SD* = 0.86) compared to females (*M* = 2.60, *SD* = 0.77), *t*(241) = -2.25, *p* = .025, *d* = 0.41. Additionally, males demonstrated a slightly higher average SWB (*M* = 3.27, *SD* = 1.08) than females (*M* = 2.89, *SD* = 1.08), *t*(241) = -1.95, *p* = .052, *d* = 0.35. Gender differences were not observed in GIBDI, flourishing, HRQoL, or the illness identity dimensions of acceptance, enrichment, engulfment, and ingroup identification.

Concerning differences between CD and UC, stronger ingroup identification was seen among CD patients (*M* = 4.79, *SD* = 2.11) compared to UC patients (*M* = 3.96, *SD* = 1.89), *t*(233) = 2.71, *p* = .007, *d* = 0.43. Moreover, CD patients reported lower average levels of SWB (*M* = 2.68, *SD* = 1.05) compared to UC patients (*M* = 3.03, *SD* = 1.10), *t*(234) = -2.02, *p* = .044, *d* = 0.32. No differences were noted between the two diseases in other variables.

**Table S6**

*Indirect Effects from Path Analysis Predicting Flourishing and Subjective Well-Being*

| Path | Unstandardized indirect effect | *SE* | Bias-corrected 95% CI |
| --- | --- | --- | --- |
| Acceptance 🠒 HRQoL 🠒 Flourishing | -0.01 | 0.02 | [-0.059, 0.020] |
| Enrichment 🠒 HRQoL 🠒 Flourishing | 0.02 | 0.02 | [-0.005, 0.075] |
| Engulfment 🠒 HRQoL 🠒 Flourishing | -0.14 | 0.05 | [-0.258, -0.057] |
| Rejection 🠒 HRQoL 🠒 Flourishing | -0.01 | 0.02 | [-0.048, 0.025] |
| Ingroup Identification 🠒 HRQoL 🠒 Flourishing | 0.00 | 0.01 | [-0.015, 0.012] |
| Acceptance 🠒 HRQoL 🠒 SWB | -0.04 | 0.05 | [-0.136, 0.064] |
| Enrichment 🠒 HRQoL 🠒 SWB | 0.06 | 0.05 | [-0.024, 0.158] |
| Engulfment 🠒 HRQoL 🠒 SWB | -0.40 | 0.06 | [-0.518, -0.303] |
| Rejection 🠒 HRQoL 🠒 SWB | -0.02 | 0.05 | [-0.114, 0.073] |
| Ingroup Identification 🠒 HRQoL 🠒 SWB | 0.00 | 0.02 | [-0.038, 0.031] |

*Note*. *N* = 243. HRQoL = Health-Related Quality of Life. SWB = Subjective Well-Being.

**Table S7**

*Indirect Effects from Path Analysis Predicting Flourishing and Subjective Well-Being Controlling for Sociodemographic and Disease Related Variables*

| Path | Indirect effect | *SE* | Bias-corrected 95% CI |
| --- | --- | --- | --- |
| Acceptance 🠒 HRQoL 🠒 Flourishing | 0.00 | 0.02 | [-0.038, 0.046] |
| Enrichment 🠒 HRQoL 🠒 Flourishing | 0.00 | 0.02 | [-0.037, 0.038] |
| Engulfment 🠒 HRQoL 🠒 Flourishing | -0.08 | 0.03 | [-0.150, -0.031] |
| Rejection 🠒 HRQoL 🠒 Flourishing | 0.00 | 0.02 | [-0.030, 0.038] |
| Ingroup Identification 🠒 HRQoL 🠒 Flourishing | 0.00 | 0.01 | [-0.011, 0.015] |
| Acceptance 🠒 HRQoL 🠒 SWB | 0.01 | 0.05 | [-0.081, 0.100] |
| Enrichment 🠒 HRQoL 🠒 SWB | 0.00 | 0.04 | [-0.083, 0.082] |
| Engulfment 🠒 HRQoL 🠒 SWB | -0.20 | 0.04 | [-0.295, -0.126] |
| Rejection 🠒 HRQoL 🠒 SWB | 0.01 | 0.04 | [-0.068, 0.081] |
| Ingroup Identification 🠒 HRQoL 🠒 SWB | 0.00 | 0.01 | [-0.025, 0.030] |

*Note*. *N* =233. HRQoL = Health-Related Quality of Life. SWB = Subjective Well-Being. Sociodemographic (age, gender, and SWB) as well as disease specific variables (IBD variant, disease duration and GIBDI) were included as controls.

**p* < .01, ***p* < .001.

Upon controlling for sociodemographic and disease-related variables, HRQoL remained a significant predictor of both flourishing and SWB, confirming H1. Moreover, both indirect effects linking engulfment to flourishing and SWB through HRQoL remained significant, providing more robust evidence for H3d. Acceptance (H3a), enrichment (H3b), rejection (H3c), and identification's (H4b) indirect effects maintained non-significance, providing partial support for H3 overall. Moreover, enrichment positively predicting and engulfment negatively predicting flourishing directly stayed significant. For SWB, only enrichment's positive direct effect remained significant in the controlled model, confirming enrichment's central role in flourishing and SWB.

**Figure S1**

*Diagram of Path Model Predicting Flourishing and Subjective Well-Being Controlling for Sociodemographic and Disease Specific Variables*

Identification

HRQoL

Flourishing
in life

Subjective
Well-Being

*R*^2^ = .53**

*R*^2^ = .59***

Engulfment

Enrichment

Acceptance

Rejection

-0.37***

0.00

0.01

0.01

0.00

-0.27***

0.24***

0.11

0.11

0.03

0.23**

-0.07

0.34***

-0.13

0.14

0.05

0.55***

0.32***

*Note*. *N* =243. Path coefficients were unstandardized estimates. Dashed lines represent nonsignificant effects. HRQoL = Health-Related Quality of Life. Covariances between predictors and covariates are included in the model but are not shown graphically. Sociodemographic (age, gender, and subjective social status) as well as disease specific variables (IBD variant, disease duration and GIBDI) were included as controls but are not depicted for parsimony.

** p* < .05, ** *p* < .01, *** *p* < .001.
